# Proteomic Analysis of Saliva in HIV-Positive Heroin Addicts Reveals Proteins Correlated with Cognition

**DOI:** 10.1371/journal.pone.0089366

**Published:** 2014-04-09

**Authors:** Stephen S. Dominy, Joseph N. Brown, Mark I. Ryder, Marina Gritsenko, Jon M. Jacobs, Richard D. Smith

**Affiliations:** 1 Department of Psychiatry, University of California San Francisco, San Francisco, California, United States of America; 2 Biological Sciences Division, Pacific Northwest National Laboratories, Richland, Washington, United States of America; 3 Division of Periodontology, Department of Orofacial Sciences, University of California San Francisco, San Francisco, California, United States of America; Max Planck Institute of Psychiatry, Germany

## Abstract

The prevalence of HIV-associated neurocognitive disorders (HAND) remains high despite effective antiretroviral therapies. Multiple etiologies have been proposed over the last several years to account for this phenomenon, including the neurotoxic effects of antiretrovirals and co-morbid substance abuse; however, no underlying molecular mechanism has been identified. Emerging evidence in several fields has linked the gut to brain diseases, but the effect of the gut on the brain during HIV infection has not been explored. Saliva is the most accessible gut biofluid, and is therefore of great scientific interest for diagnostic and prognostic purposes. This study presents a longitudinal, liquid chromatography-mass spectrometry-based quantitative proteomics study investigating saliva samples taken from 8 HIV-positive (HIV^+^), 11 −negative (HIV^−^) heroin addicts. In addition, saliva samples were investigated from 11 HIV^−^, non-heroin addicted healthy controls. In the HIV^+^ group, 58 proteins were identified that show significant correlations with cognitive scores, implicating disruption of protein quality control pathways by HIV. Notably, only one protein from the HIV^−^ heroin addict cohort showed a significant correlation with cognitive scores, and no proteins correlated with cognitive scores in the healthy control group. In addition, the majority of correlated proteins have been shown to be associated with exosomes, allowing us to propose that the salivary glands and/or oral epithelium may modulate brain function during HIV infection through the release of discrete packets of proteins in the form of exosomes.

## Introduction

HIV-associated neurocognitive disorders (HAND) remain common, even among patients receiving combination antiretroviral therapy (cART) [1]. The mechanisms underlying HAND remain elusive, but a history of a low nadir CD4^+^ count has been shown to be a strong predictor of cognitive impairment before and after the cART era [1], [2]. In addition, it has been proposed that heroin and other illicit drugs are cofactors in HAND [3]. One area that has not been investigated in relation to HAND is the influence of the gut and associated microbiome on neurocognition. Evidence has emerged over the last decade that gut immune, neural, and enteroendocrine mechanisms as well as soluble factors or metabolites of gut commensal bacteria may influence the brain [4]–[7]. However, the specific pathways and molecules underlying communication between the gut and brain are poorly understood. An enhanced understanding of gut proteins, both human and microbial, underlying gut-brain interactions during HIV infection in heroin addicted persons may provide novel insights into HAND in this population. This study focused on human proteins in saliva and their relationship to cognition, as saliva is one of the most clinically accessible gut biofluids, and accumulating evidence suggests that the study of salivary proteins may provide insight into the pathophysiological mechanisms of neurodegenerative diseases [8]–[12]. Previous proteomic studies of cognition in HIV disease have analyzed cerebrospinal fluid (CSF) [13], [14], serum [15], [16], macrophage secretions [17], and brain tissue [18], [19] in non-heroin addicted subjects. Rozek et al. identified twenty differentially expressed proteins in CSF of HIV^+^ subjects with and without HIV-associated dementia (HAD), including six proteins they validated by western blot analysis (cystatin C, gelsolin, complement C3, clusterin, vitamin D-binding protein, and procollagen-C –endopeptidase enhancer 1) [13]. Laspiur et al. identified a set of CSF proteins associated with cognitive impairment in HIV disease that included soluble superoxide dismutase (SOD 1), migration inhibitory factor (MIF)-related protein 14, macrophage capping protein, neurosecretory protein VGF, galactein-7, L-plastin, acylphosphatase 1, and tyrosine 3/tryptophan 5-monoxygenase activation protein [14]. Proteomic analysis of serum in HAD by Wiederin et al. revealed gelsolin and prealbumin as differentially expressed proteins [15]. A novel study by Toro-Nieves et al. analyzed proteomes of macrophages infected by HIV-1 viral strains from HIV^+^ women with cognitive impairment or normal cognition. Toro-Nieves' group found distinct changes in macrophage proteins between the two conditions, with macrophages infected with HIV-1 strains from the cognitively impaired subjects demonstrating changes in proteins related to apoptosis, chemotaxis, inflammation, and redox metabolism [17].

In the first proteomic study to examine autopsied brain tissue from subjects with HAD and subjects with HIV infection but without dementia, Zhou et al. found a significant alteration of 31 proteins in HAD subjects' brain tissue compared to the non-demented, HIV-infected subjects, with 90% of the altered proteins overlapping with proteins previously identified in non-viral neurodegenerative disorders [18]. Zhou's team validated four of the altered proteins (carbonic anhydrase 2, glutamine synthetase, creatine kinase ubiquitous mitochondrial, dihydropyrimidinase-related protein 2) in brain tissue using Western blotting and immunohistochemistry [18]. Gelman and Nguyen conducted a postmortem proteomic analysis of human frontal neocortex synaptosomes from HIV-infected subjects and found altered concentrations of synapsin 1b, 14-3-3 zeta, 14-3-3 epsilon, and stathmin in the nerve endings, which the authors suggested supports an HIV-induced disruption of the ubiquitin-proteasome system (UPS), the physiological regulator of protein quality control in the synaptic compartment [19].

Disruption of the UPS in synapses has also been demonstrated for drugs of abuse. Stockton and Devi recently conducted a review of proteomic studies looking at the morphine-dependent synapse, and found morphine consistently induces significant changes in proteins in the UPS, with heat shock and heat shock-related proteins over represented in neuroproteomic studies of morphine [20]. Wang et al. recently reviewed proteomic studies of addictive drugs and noted 497 proteins that have been associated with drug exposure, including many proteins involved in synaptic transmission and signaling pathways [21]. Recently, Brown et al. administered morphine to two species of nonhuman primates over a 20-day period and measured protein changes in inguinal lymph node, colon, peripheral blood, and CSF [22]. Morphine exposure resulted in altered protein expression in all tissues examined in both species, with a significant alteration of the expression of 107 proteins in the CSF of African green monkeys, and 17 proteins in the CSF of pigtailed macaques. Functional categories of CSF proteins altered by morphine in the NHPs included immune response, cell growth, transport, energy metabolism, and signal transduction [22]. In human opiate addicts, Zill et al. performed postmortem proteomic analysis on brain tissue from the amygdala and found alterations in the expression of beta-tubulin and heat-shock protein HSP60 when compared to controls [23].

Drug-induced changes in protein expression may be responsible, in part, for the finding that drug addiction can lead to cognitive deficits across multiple domains, especially cognitive function related to frontal-striatal circuitry [24]. Heroin addiction in particular has been linked to gray matter deficits [25]–[27] and accelerated brain aging [28]. Methadone, an opioid treatment for heroin addiction, has also been associated with impaired cognitive function [29]–[31]. Despite these well-recognized negative effects of drugs on cognition, Byrd et al. recently demonstrated that the cognitive effects of HIV can be isolated in drug-addicted, HIV-infected cohorts [24].

We were thus motivated to take a prospective, longitudinal, and unbiased approach using proteomics to discover correlations that might exist between human salivary proteins and cognition in HIV^+^ and HIV^−^ heroin addicts during the initial phases of methadone treatment, and to use a group of healthy, HIV^−^, non-addicted subjects as a control. The focus of our study was to investigate a set of fundamental questions concerning salivary protein abundances and their correlation with cognition, particularly in the context of heroin and polydrug use in HIV^+^ and HIV^−^ subjects. The approach undertaken involved submitting serial saliva samples to high mass accuracy and high throughput liquid chromatography based mass spectrometry (LC-MS/MS), which would allow, within subjects, an unbiased view of the proteins shed/secreted into this clinical biofluid and allow correlational analyses between salivary protein abundances and serial cognitive scores as measured by the Digit Symbol Substitution Test (DSST) [32], [33]. The overall driving analytical questions include, what, if any, correlations can be seen between the abundance of specific salivary proteins and DSST scores in HIV^+^ or HIV^−^ heroin addicts, how these proteins are related longitudinally to methadone treatment and HIV status, and can any of these identified proteins be used as potential indicators of neurodegenerative disease mechanisms.

## Materials and Methods

### Ethics Statement

The study was approved by the University of California Committee on Human Research and each participant provided voluntary written informed consent.

### Research Participants

Participants were drawn from the methadone maintenance clinic at San Francisco General Hospital upon presentation for treatment of heroin addiction. Eight participants were HIV^+^ and eleven participants were HIV^−^. Healthy, non-addicted HIV^−^ participants (*n* = 11) were drawn from a control group for a drug interaction study [34]. Table 1 presents data on demographic characteristics for all study participants. There were significantly more women among the HIV^−^ subjects relative to the HIV^+^ subjects, and the HIV^−^ subjects were using significantly more methamphetamine/amphetamine than HIV^+^ subjects.

**Table 1:** Demographics.

	Heroin Dependent, HIV^−^ N = 11	Heroin Dependent, HIV^+^ N = 8	Non-Heroin, HIV^−^ N = 11
Age (yrs)^1^	40.00 (2.97)	42.38 (2.96)	43.27 (2.97)
Weight (kg)	73.22 (4.48)	72.16 (3.28)	78.8 (4.2)
Female^2^	5 [45%]^3^	0 [0%]	3 [27.2%]
Race:			
African-American	1 [9%]	2 [25%]	5 [45.4%]
White	7 [64%]	6 [75%]	6 [54.5%]
Other	3 [27%]	0 [0%]	
Other Substance Use:			
Amphetamines	7 [64%]^3^	1 [13%]	0 [0%]
Benzodiazepines	5 [45%]	4 [50%]	0 [0%]
Cocaine	6 [55%]	7 [88%]	0 [0%]
Marijuana	7 [64%]	6 [75%]	1 [9.0%]
Nicotine Use (packs/day)	.83 (.14)	.98 (.25)	0.07 (0.06)
Viral Load	N/A	9281.25 (8915.30)	N/A
CD4	N/A	723.75 (221.64)	N/A
cART Exposure	N/A	7 [88%]	N/A
Hepatitis C Infection	10 [91%]	8 [100%]	0 [0%]

1 Mean (SE).

2 [ ] percent of sample affected.

3 p = 0.04.

### Serial Saliva Sample Collection and Cognitive Testing

Saliva samples were collected by having participants chew on a small square of Parafilm® (Menasha, WI) for five minutes or less and spit into a sterile collection cup. Saliva samples were then aliquoted and frozen at −80°C until time of analysis. Saliva sample collection and cognitive testing with the 2-minute computerized version of the DSST [35] were performed concurrently on five separate occasions; the first sampling session occurred on day 1 when the participant first presented to the methadone clinic and before the participant received their first dose of methadone; the second and third sampling sessions occurred on average on days 35 and 36, respectively; and the fourth and fifth sampling sessions occurred on average on days 100 and 102, respectively. All samples were processed individually, and a minimal number of time points were missed by some participants. For the control group, saliva sampling and cognitive testing with the DSST were performed on two separate occasions separated by a period of 15 days. The computerized version of the DSST asks subjects to match symbols with their corresponding digit within a two minute time limit. The number of correct responses within the two minute time limit is a measure of executive cognitive functioning, working memory, processing speed, and visuo-spatial attention [36]. There is not an agreed-upon threshold for normality for the DSST score, but lower DSST scores indicate worse performance [36]. At each sampling time point, urine was tested for recent use of illicit opioids, cocaine, amphetamines, benzodiazepines, and marijuana.

#### ELISA analyses

Samples were prepared for ELISA analysis by removing mucin precipitate and other particulate matter by centrifuging samples at 1500×*g* for 15 min. A commercially available IL1RA ELISA kit (R&D Systems DRA-00B) was used to measure the concentration of IL1RA samples in saliva from HIV^+^ subjects. The protocol supplied with the ELISA kit was followed, except that Saliva Matrix (Salimetrics LLC, 3–3000) was used as a diluent in place of the kit-provided assay diluents to better normalize the protein antibody binding conditions for saliva samples.

#### Sample preparation for proteome analysis

Saliva samples were denatured with the addition of solid urea to a final concentration of 8 M. Protein concentration was determined by bicinchoninic acid assay (Pierce Biotechnology, Inc., Rockford, IL, USA). To enhance proteolysis, samples were reduced with 10 mM DTT for 1 hour at 37°C. For trypsin digestion, samples were diluted 10-fold with 50 mM NH_4_HCO_3_, pH 7.8, and digestion was performed with sequencing grade-modified porcine trypsin prepared according to the manufacturer (Promega, Madison, WI, USA). Saliva proteins were digested at 1∶50 (wt/wt) trypsin-to-protein ratio for 3 hours at 37°C, and desalted by solid-phase extraction (Discovery DSC-18, SUPELCO, Bellefonte, PA). Peptides were eluted from the SPE column using 80% acetonitrile with 0.1% trifluoroacetic acid, and dried in Speed-Vac SC 250 Express (Thermo Savant, Holbrook, NY).

#### RPLC separation and MS(/MS) acquisition

The method used in this study, with the coupling of a constant-pressure (5,000-lb/in^2^) reversed-phase capillary liquid chromatography (RPLC) system (75-µm inside diameter, 360-µm outside diameter, 65-cm capillary; Polymicro Technologies Inc., Phoenix, AZ, USA) and a Velos LTQ-Orbitrap mass spectrometer (MS; Thermo Scientific, Waltham, MA, USA) using an electrospray ionization source manufactured in-house, has been previously reported [37]–[39]. The instrument was operated in data-dependent mode with an m/z range of 400–2000. The 10 most abundant ions from the MS analysis were selected for MS/MS analysis using a normalized collision energy setting of 35%. A dynamic exclusion of 1 min was used to avoid repetitive analysis of the same abundant precursor ion. The heated capillary was maintained at 200°C, and the ESI voltage was held at 2.2 kV [37]–[39].

#### Mass spectrometry data analysis

The LC-MS/MS raw data were converted into a .dta file using an in-house software, DeconMSn (version v2.1.4.1), which accurately calculates the parent monoisotopic mass for each spectrum from the parent isotopic distribution using a modified THRASH approach. The SEQUEST software ((Version v.27, Rev 12, Thermo Fisher Scientific, Waltham MA)) was used to search the MS/MS spectral data against human UniProt fasta file containing 20,276 protein sequences, downloaded May 5, 2011. Porcine trypsin was added into the database as an expected contaminant. No cleavage specificity was defined in the database searching. For AMT tag database creation, raw SEQUEST results were filtered on: (1) Xcorr ≥1.9 for charge state +1, (2) Xcorr ≥2.2 for charge state +2, and (3) 3.75 for charge state +3, using 3.0 and 1.0 Da for parent and fragment ion tolerances, respectively. High resolution LC-MS features were deconvoluted by Decon2Ls (version 1.0.2, using default parameters) and aligned to the mass and time tag database using VIPER (version 3.45 using default parameters) using the theoretical mass and observed normalized elution times (NET) for each peptide. This approach to proteomics research is enabled by a number of both published [40]–[44] and unpublished in-group development tools that are freely available for download at http://omics.pnl.gov. Peptide alignment results were further refined with a FDR<5% based on STAC and uniqueness probability measurements [45], mass error <2 ppm, and peptide identifications were filtered using a MSGF score <1E-10 [46].

### Statistical analysis

All statistical analyses were performed with the R software framework [47]. Peptide relative abundance values were Log_2_ transformed and used for an ANOVA analysis within DanteR [48](http://omics.pnl.gov). Relative protein abundance was estimated by using median peptide intensity values [49], requiring a minimum of 2 unique peptides. Pearson product-moment correlations were performed to determine the strength of linear dependence between DSST scores and relative protein abundances. *P* values from ANOVA and Pearson correlation analyses were corrected for multiple tests using the Benjamini-Hochberg adjustment [50]. Proteins were considered to have shown a significant change in salivary abundance with *P*≤0.05.

## Results

**Figure 1:**
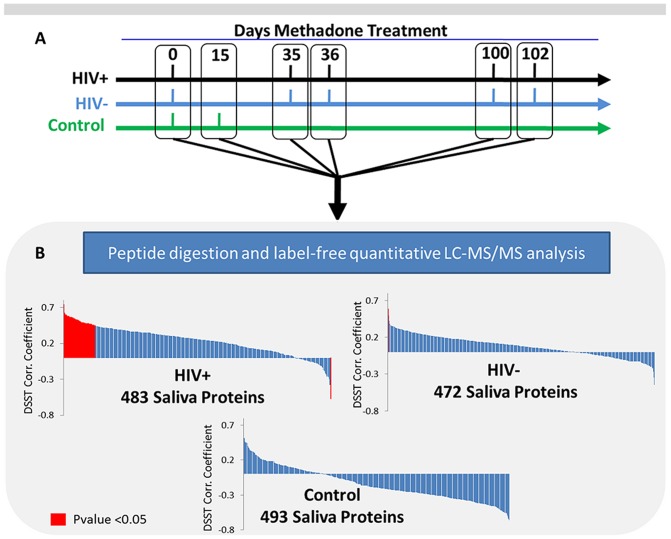
Overview of clinical saliva sample collection and proteomic analysis. A) Timeline of the three participant cohorts and their respective days of saliva sample collection and DSST testing. The control population was HIV^−^ and heroin independent so no methadone treatment was initiated at day 0. B) Graph of DSST correlation with all identified and quantified saliva proteins from each respective group after sample collection, processing, instrument and data analysis. Proteins shown in red are those which pass the Benjamini-Hochberg corrected *P*-value of <0.05.

The objective of this study is to determine if significant correlations exist between the abundance of specific salivary proteins and DSST cognitive scores from HIV^+^ or HIV^−^ individuals receiving treatment for heroin addiction. The proteomic profile of eight HIV^+^, and eleven HIV^−^ heroin dependent subjects (at five longitudinal time points) were included, along with 11 HIV^−^, non-heroin dependent subjects as controls (at two time points), see Table 1 and Figure 1A. Starting at day 0, heroin dependent subjects initiated methadone treatment, which continued throughout the study. Across all five time points during the course of the study, whole saliva was collected for proteomic analysis and a DSST score of cognition was determined (See Table S1). LC-MS/MS analyses and label-free quantitative data analysis (see materials and methods for details) identified and quantified the abundance of 472 salivary proteins in the HIV^−^ group, 483 proteins in the HIV^+^ group, and 493 proteins in the HIV^−^, heroin independent group, see Figure 1B and Tables S2, S3. Correlation of DSST scores with each individual protein value revealed only a single significant protein correlation in the HIV^−^ group, in contrast to 58 significant protein correlations in the HIV^+^ group (*P*<0.05) (Table 2). No significant DSST score/saliva protein correlation was observed in the HIV^−^, heroin independent control group. Table S4 provides the overlap of salivary proteins from our current study that correlate with DSST scores and proteins that have been shown by other investigators to be modulated by morphine and other drugs of abuse. In addition, Table S5 provides the overlap of salivary proteins with correlations of DSST scores and proteins identified in prior proteomic studies related to HIV infection.

**Table 2:** Proteins showing a significant correlation between abundance and DSST score.

UniProt	Name	Symbol	Corr	P-value
P27824	calnexin	CANX	0.74	9.96E-05
P61158	ARP3 actin-related protein 3 homolog (yeast)	ACTR3	0.62	0.00705
Q13885	tubulin, beta 2A class IIa	TUBB2A	0.61	0.00705
P16152	carbonyl reductase 1	CBR1	0.6	0.00841
P08107	heat shock 70 kDa protein 1A	HSPA1A	0.6	0.00841
Q13509	tubulin, beta 3 class III	TUBB3	0.59	0.00912
P35579	myosin, heavy chain 9, non-muscle	MYH9	0.58	0.00912
Q3ZCM7	tubulin, beta 8 class VIII	TBB8	0.58	0.00912
O75083	WD repeat domain 1	WDR1	0.58	0.00912
P46940	IQ motif containing GTPase activating protein 1	IQGAP1	0.57	0.00912
Q14134	tripartite motif containing 29	TRIM29	0.57	0.00912
P07437	tubulin, beta class I	TUBB	0.57	0.00912
Q8TD99	desmin	DES	0.56	0.00912
A6NNZ2	tubulin beta-8 chain B	TBB8B	0.56	0.00912
A6NKZ8	putative tubulin beta chain-like protein	YI016	0.56	0.00912
P18510	interleukin 1 receptor antagonist	IL1RA	−0.57	0.00912
P21333	filamin A, alpha	FLNA	0.56	0.00924
Q6PEY2	tubulin, alpha 3e	TUBA3E	0.56	0.00924
Q96ML2	vimentin	VIM	0.55	0.00924
Q9H4B7	tubulin, beta 1 class VI	TUBB1	0.54	0.0125
P60842	eukaryotic translation initiation factor 4A1	EIF4A1	0.54	0.0128
P63244	guanine nucleotide binding protein (G protein), beta polypeptide 2-like 1	GNB2L1	0.54	0.0133
P11413	glucose-6-phosphate dehydrogenase	G6PD	0.53	0.0143
P07900	heat shock protein 90 kDa alpha (cytosolic), class A member 1	HSP90AA1	0.53	0.0143
Q9BYX7	POTE ankyrin domain family, member I	POTEKP	0.53	0.0143
P55072	valosin containing protein	VCP	0.53	0.0143
P15104	glutamate-ammonia ligase	GLUL	0.52	0.016
P14780	matrix metallopeptidase 9	MMP9	0.52	0.0162
P59998	actin related protein 2/3 complex, subunit 4, 20 kDa	ARPC4	0.51	0.0223
Q71UI9	H2A histone family, member V	H2AFV	0.51	0.0223
P00491	purine nucleoside phosphorylase	PNP	0.5	0.0227
O15231	zinc finger protein 185 (LIM domain)	ZNF185	0.5	0.0232
P80188	lipocalin 2	LCN2	0.5	0.0271
P62269	ribosomal protein S18	RPS18	0.49	0.0286
P17931	lectin, galactoside-binding, soluble, 3	LGALS3	0.49	0.0291
P35749	myosin, heavy chain 11, smooth muscle	MYH11	0.49	0.0291
Q9H882	myosin-14	MYH14	0.49	0.0291
P62244	ribosomal protein S15a	RPS15A	0.49	0.0303
P35580	myosin, heavy chain 10, non-muscle	MYH10	0.48	0.0309
P15153	ras-related C3 botulinum toxin substrate 2	RAC2	0.48	0.031
P26641	eukaryotic translation elongation factor 1 gamma	EEF1G	0.48	0.0327
P02042	hemoglobin, delta	HBD	0.48	0.0327
P05164	myeloperoxidase	MPO	0.48	0.0327
Q6S8J3	POTE ankyrin domain family, member E	POTEE	0.48	0.0327
O15145	actin related protein 2/3 complex, subunit 3, 21 kDa	ARPC3	0.48	0.0332
P02144	myoglobin	MB	0.47	0.0338
P43490	nicotinamide phosphoribosyltransferase	NAMPT	0.47	0.0338
P02511	crystallin, alpha B	CRYAB	0.47	0.0367
A5A3E0	POTE ankyrin domain family, member F	POTEF	0.47	0.0367
P29401	transketolase	TKT	0.47	0.0367
P22314	ubiquitin-like modifier activating enzyme 1	UBA1	0.47	0.0367
P30613	pyruvate kinase, liver and RBC	PKLR	0.46	0.0386
P12814	actinin, alpha 1	ACTN1	0.46	0.0406
P08238	heat shock protein 90 kDa alpha (cytosolic), class B member 1	HSP90AB1	0.46	0.0406
Q549N7	hemoglobin subunit beta	HBB	0.45	0.0425
P19105	myosin, light chain 12A, regulatory, non-sarcomeric	MYL12A	0.45	0.0425
Q86Y46	keratin 73	KRT73	0.45	0.0449
P01877	Ig alpha-2 chain C region	IGHA2	0.45	0.0467

### Proteins Involved in Protein Quality Control Have Positive Correlations with Cognitive Scores

**Figure 2:**
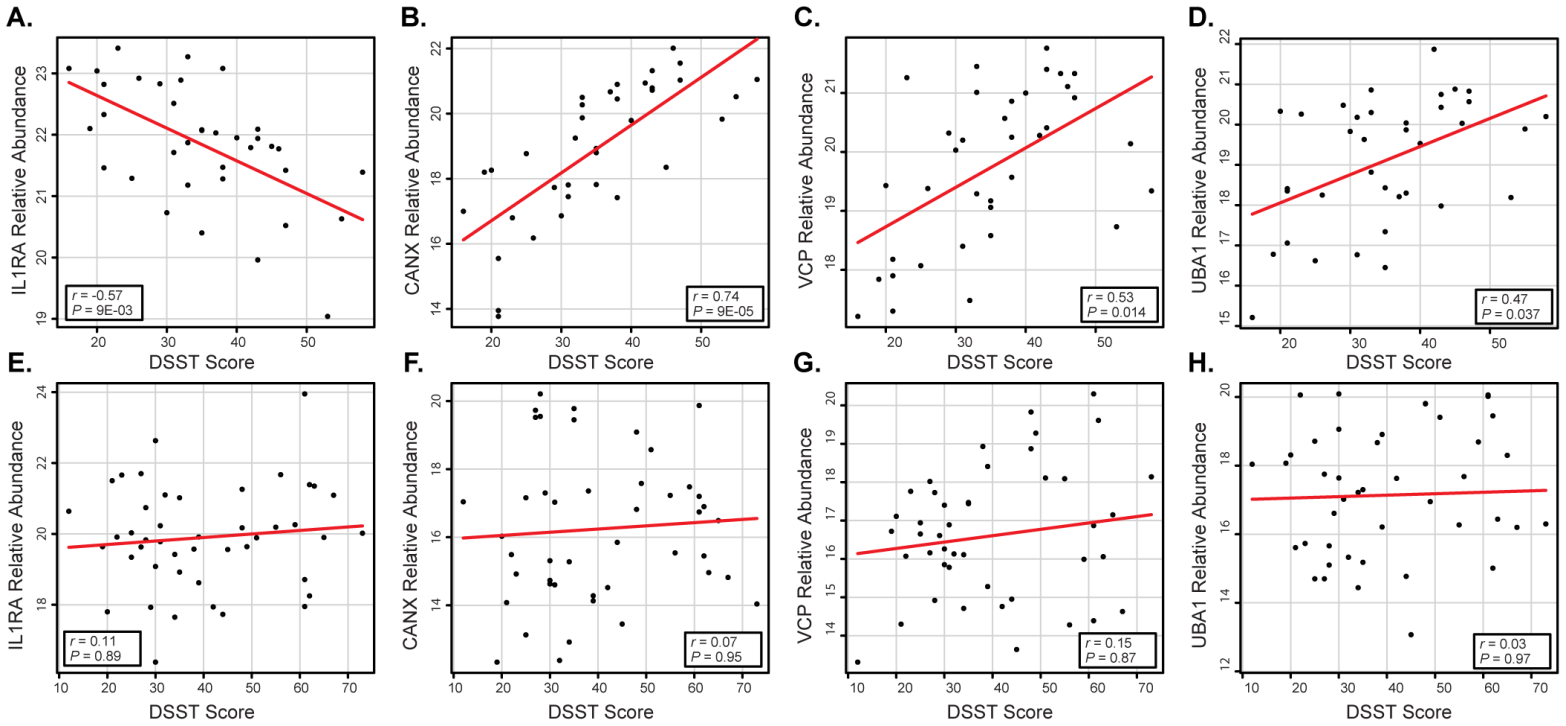
Differences in HIV^+^ vs. HIV^−^ scatter plots of DSST/protein abundance correlation analyses results for four salivary proteins of interest. (A–D) Scatter plots showing the correlation of specific salivary protein abundance values against measured DSST scores in HIV^+^, heroin dependent individuals. The proteins include IL1RA (interleukin-1 receptor antagonist), CANX (calnexin), VCP (valosin-containing protein), and UBA1 (ubiquitin-like modifier-activating enzyme 1). All proteins, except for IL1RA, show significant direct correlation of DSST with salivary protein abundance at *P-*value<0.05. (E–H) Scatter plots showing the same comparison except against HIV^−^ heroin dependent individuals; however, no perceived correlation is observed, i.e., all p-values are well above 0.05. The linear regression is shown by a red line, and the Pearson product-moment correlation score (*r*) and Benjamini-Hochberg corrected *P*-values are indicated in the inlay boxes.

Multiple saliva proteins of interest were observed to have a correlative relationship with DSST scores, specifically, a significant positive correlation was determined between salivary valosin-containing protein (VCP, aka TERA/p97/Cdc48) and DSST scores (r = 0.53 and *P* = 0.014), see Figure 2, in HIV^+^ heroin addicts. VCP is a key component in the cytosolic degradation of proteins by the proteasome and it also plays a key role in autophagy [51]. One of the main functions of VCP involves the disassembly of protein complexes and aggregates by extracting ubiquitinated proteins from both the endoplasmic reticulum and the outer mitochondrial membrane and presenting the ubiquitin-tagged proteins to the proteasome for degradation [51]–[53]. Disruption of this pathway has been found to lead to excessive accumulation of ubiquitinated proteins and cellular and mitochondrial dysfunction [53]. Watts et al. found that mutations in the VCP gene result in the autosomal-dominant degenerative disease known as Inclusion Body Myositis with Paget's disease of the bone and Frontotemporal Dementia [54]. VCP has also been linked to Huntington's disease, Parkinson's disease, amyotrophic lateral sclerosis, and Alzheimer's disease [51], [52], [55]. Of note, Jager et al. recently demonstrated a direct protein-protein interaction between HIV gp41 and VCP [56].

Salivary ubiquitin-like modifier-activating enzyme 1 (UBA1, aka UBE1) was also observed with a significant positive correlation with cognitive scores, as measured by the DSST, in HIV^+^ heroin addicts (r = 0.47 and *P* = 0.036) (Figure 2). UBA1 is critical for protein quality control since it is involved in the first step of activating ubiquitin to mark damaged proteins for degradation by the proteasome [57]. Mutations in the UBA1 gene have been found to alter the ubiquitin-proteasome system resulting in an early-onset, fatal neurodegenerative disorder involving lower motor neurons in childhood [58]. Jager's group has demonstrated a direct protein-protein interaction between UBA1 and the HIV integrase protein [56].

A third protein critically involved in protein quality control that was found to be significantly correlated with DSST scores is calnexin (CANX)(r = 0.74 , *P* = 9.96E-05) (Figure 2). Calnexin is a transmembrane chaperone of the endoplasmic reticulum that ensures the proper folding and quality control of newly synthesized glycoproteins [59], [60]. *In vitro* studies have demonstrated that calnexin may be important in protecting against neurotoxicity associated with misfolded proteins [61]. HIV gp160 has been shown to have a direct protein-protein interaction with calnexin [56], [62].

### Interleukin-1 Receptor Antagonist Has a Negative Correlation with Cognitive Scores

Strikingly, only one salivary protein demonstrated a significant negative correlation with DSST scores, IL-1 receptor antagonist (IL1RA) (r = −0.57, *P* = 0.009) (Figure 1). IL1RA is a naturally occurring antagonist of IL-1 that is capable of blocking IL-1 effects by binding to its receptor [63]. HIV has been shown to induce an excessive abundance of IL1RA when compared to IL-1 in human primary monocytes *in vitro*. Zavala et al. demonstrated that HIV triggered IL1RA in a mean 1,000-fold increase over IL-1αβ, a ratio 20-fold higher than that obtained with lipopolysaccharide [64]. IL1RA is associated with several neurodegenerative disorders (see Discussion below).

### Detection of Salivary IL1RA by ELISA

**Figure 3:**
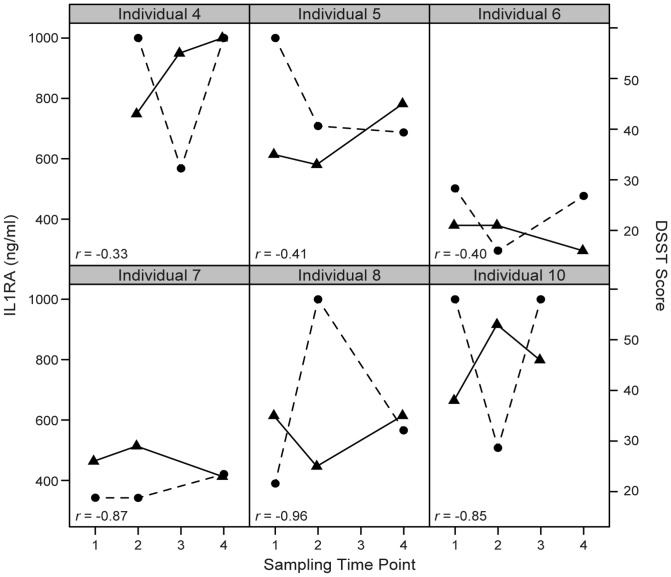
Inverse relationship of IL1RA ELISA results with DSST scores. Scatter plot of IL1RA ELISA absolute concentrations (circles and dotted lines) and DSST scores (triangles and solid lines) over time across 6 HIV-positive individuals showing consistent inverse correlation. Sampling time points include 1 (the day participant presents to the methadone clinic and begins first dose), 2 and 3 (second and third sessions ∼35 and 36 days of methadone, respectively), and 4 (∼100 days on methadone). The Pearson product-moment correlation scores are shown in the lower left corner of each scatter plot.

For the reasons described above and to demonstrate that the inverse relationship between IL1RA and DSST can be detected by alternative quantitative strategies, this receptor antagonist was selected for targeted analysis by ELISA. IL1RA concentrations, which ranged from 290 ng/ml to >1 µg/ml, were measured in 6 HIV^+^ individuals across 4 time points during the study. Similar to the LS-MS/MS results, DSST scores and the absolute concentrations of IL1RA demonstrate an inverse correlation; see Figure 3, further supporting initial quantitative observations by LC-MS/MS.

### Heat Shock Response Proteins are Positively Correlated with Cognitive Scores

Significant positive correlations between protein abundance and DSST scores were identified with 4 important heat shock proteins: heat shock 70 kDa protein 1A/1B (HSPA1A)(r = 0.6, *P* = 0.008), heat shock protein HSP 90-alpha (HSP90AA1)(r = 0.53, *P* = 0.014), alpha-crystallin B chain (CRYAB/HSPB5)(r = 0.47, *P* = 0.036), and heat shock protein HSP 90-beta (HSP90AB1)(r = 0.46, *P* = 0.040) (Table 2). The heat shock protein response is a highly conserved physiologic mechanism that protects cells against the cytotoxic effects of protein aggregation [65]. Heat shock proteins are molecular chaperones that assist in the folding of misfolded proteins, preventing their aggregation [66]–[69]. The Hsp90 paralogues, HSP90AA1 and HSP90AB1, occupy central positions in protein homeostasis [70], [71].

### Differential Saliva Protein Abundances due to Methadone Treatment

**Figure 4:**
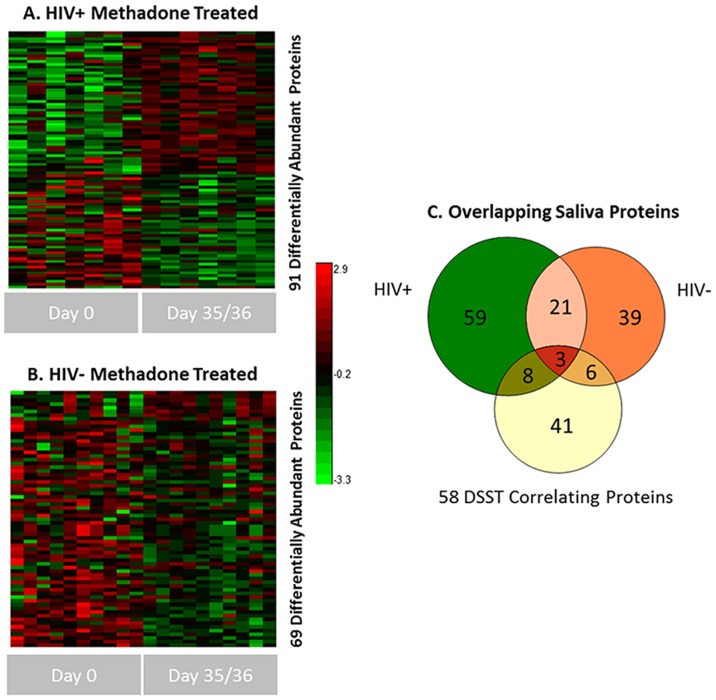
Overview of differentially abundant saliva proteins across methadone treatment. A) and B) Heat map of all differential protein abundances in the HIV^+^ and HIV^−^, heroin dependent cohorts respectively due to methadone treatment (see supplemental table 3 for complete list) at p value <0.05. Clustering is based upon a Pearson distance metric with K-means clustering (K:5). Protein abundance values were scaled for plotting. C) Venn diagram showing the overlap between the respective HIV ^+/−^ heat map results and the previous 58 DSST correlating saliva proteins.

Due to the longitudinal nature of the study, we performed a comparative statistical analysis of the overall saliva protein abundance differences observed due to methadone treatment for both HIV^+^ and HIV^−^ individuals. Comparisons were made between time point 0 (time of first treatment) and 35/36 days into treatment, see Figure 4A and 4B. These sampling points provided the most direct comparison for determining a methadone treatment effect. 91 and 69 saliva proteins were observed as differentially abundant across the HIV^+^ and HIV^−^ individuals respectively (see table S6). The overlap between each saliva protein subset and those proteins previously observed as correlative with DSST scores is shown in Figure 4C. Initial observations include a somewhat more homogenous set of differential proteins apparent in the HIV^+^ subset compared to the HIV^−^ cohort, and interestingly a minimal overlap with DSST correlative proteins. Based upon such observations, we can now preliminarily discriminate those protein abundance changes which are likely to be dependent or independent (to some extent) of HIV status and/or methadone treatment. Both VCP and Calnexin overlap with methadone treatment protein changes and would appear to be sensitive to such perturbations, but only in the HIV^+^ set, while IL1RA, UBA1, and all altered heat shock proteins do not overlap and appear to be unrelated to methadone treatment effects. Additionally, since there was no change in HIV status within this timeframe, we would not necessarily expect a high overlap of DSST correlative proteins with the HIV^+^ group, however proteins uniquely identified as differential between HIV^+^ and HIV^−^ individuals are likely due to HIV status and/or cART treatment. Conversely, those 24 proteins (21+3) which do overlap between HIV^+^ and HIV^−^ individuals can be viewed as saliva proteins changing independent of HIV status, and hence more related specifically to methadone treatment.

## Discussion

Using a high mass accuracy LC-MS/MS based proteomic analysis, we found significant correlations between salivary proteins and DSST cognitive scores in HIV^+^ heroin addicts. Importantly, we found only one correlation between salivary proteins and cognitive scores in HIV^−^ heroin addicts, and no correlations in HIV^−^ heroin independent individuals. Additionally, the effect of methadone treatment across both HIV^+^ and HIV^−^ individuals informed upon the possible links of DSST correlative proteins to the sensitivity to methadone treatment. As noted above, the salivary proteins in HIV^+^ heroin addicts that correlate with cognitive scores implicate molecular mechanisms involved in protein quality control and neurodegenerative diseases. Our proteomic results support Kragh et al.'s recent suggestion that defective protein quality control underlies HAND pathogenesis [72]. Kragh's group proposed that protein quality control could be disrupted if HIV proteins bound directly to components of the protein quality control machinery [72]. Jager et al. recently demonstrated that HIV proteins do bind directly to components of the protein quality control machinery, including VCP, UBA1,and CANX, proteins that we observed in the saliva of HIV^+^ heroin addicts to have significant correlations with cognitive scores [56] (Table 2). Additional evidence of disrupted protein quality control in our current study comes from the significant correlations between the heat shock proteins HSPA1A, HSP90AA1, CRYAB, HSP90AB1 and cognitive scores (Table 2).

The question arises as to what type of mechanism could account for the significant correlations between multiple salivary proteins and cognitive scores in HIV^+^ heroin addicts. During analysis of the salivary protein dataset, we observed that many of the identified proteins are common exosomal markers, for example, the heat shock protein HSP90AA1 is one of the most common exosomal markers in the ExoCarta database [73]. Exosomes are 30–100 nm membrane vesicles of endocytic origin released from multivesicular bodies into the extracellular environment by most cells [73]. Proteomic studies have shown that certain proteins are common to all exosomes, including chaperones and cytoskeletal proteins, regardless of their cell of origin, while other exosomal proteins are cell-type specific [74]. In addition to protein, exosomes contain mRNA and miRNA that can be shuttled from one cell to another, altering gene expression in the receiving cell [75]. Of note, the salivary glands produce large amounts of exosomes [76]. Of particular relevance, proteomic analysis of the salivary parotid exosome by Gonzalez-Gegne et al. revealed numerous proteins associated with neurodegenerative disorders and prion disease [74]. Recent proof-of-concept studies have demonstrated that exosomes originating in the periphery can cross the blood brain barrier and influence neuronal functions [77], [78]. Zhuang et al. have suggested that exosomes from the periphery may play a role in brain function [77].

### HIV and the Trojan Exosome Hypothesis

The possible role of exosomes in HIV disease was first proposed by Gould et al. in 2003 [79]. Gould's group hypothesized that HIV, and other retroviruses, use the preexisting cellular exosome biogenesis pathway to form infectious exosomes that can be taken up by other cells independent of Env proteins and receptors [79]. However, the hypothesis has been challenged, with evidence showing that HIV-1 can be budded from CD4+ T-lymphocytes independently of exosomes [80]. Other researchers have provided evidence that HIV virion preparations may be contaminated by cellular exosomes [81].

Recently, Narayanan et al. conducted experiments showing that HIV-1 infected cells can produce exosomes containing unique proteins and RNA molecules, but not whole virus [82]. Narayanan's team found proteomic evidence of the HIV gp160 protein in exosomes from HIV infected cells, leading them to suggest that exosomes containing gp160 could contribute to HIV-induced neurologic damage by crossing the blood-brain barrier independent of an actual infection [82]. In addition, Hu et al. have shown that astrocytes treated with morphine and HIV Tat release exosomes containing miR-29b that can be taken up by neurons resulting in neuronal death [83]. In other, non-HIV related neurodegenerative diseases, evidence is accumulating that exosomes may be involved in the spread of neurotoxic proteins [84]. On the contrary, many studies indicate that exosomes serve a protective role in the nervous system, providing stress-protective proteins and other factors for trophic support of neurons during normal and pathological conditions [84], [85].

**Figure 5:**
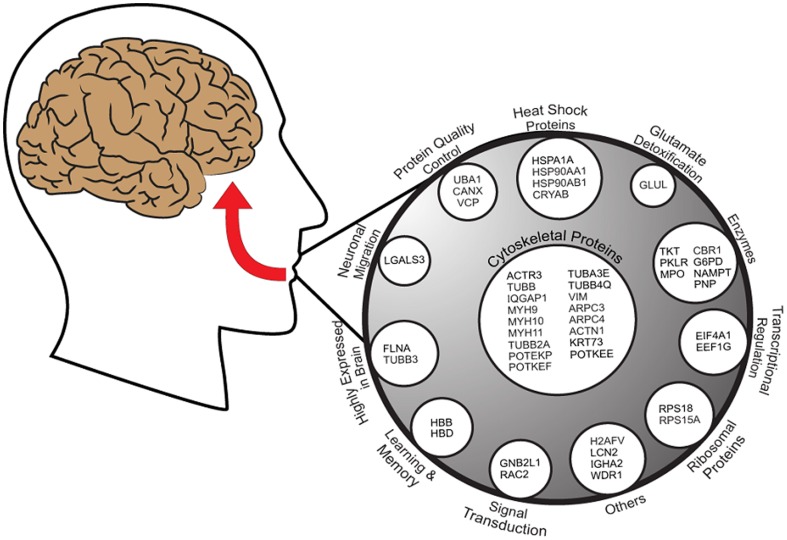
A graphical representation of the overlap between 47 salivary proteins that demonstrated significant positive correlations with DSST scores in HIV^+^ heroin addicts and exosomal proteins identified in the ExoCarta database [73]. Proteins are grouped by functional categories for visualization purposes, and not sub-exosomal localization. Adapted from Mathivanin et al. [105].

We therefore compared our list of salivary proteins that exhibit significant correlations with cognitive scores with the list of exosomal proteins contained in the ExoCarta database [73], and found that 48 of the 58 salivary proteins (82%) have been identified as components of exosomes [73]. Figure 5 is a schema of the 47 salivary proteins with positive correlations, organized by function, that have been identified in exosomes. IL1RA, the only salivary protein in our data set to exhibit a significant negative correlation with cognitive scores, has also been identified in exosomes [73].

Interestingly, several proteins involved in neuronal protection and regeneration appear in our dataset, including LGALS3 (neuronal migration and axon branching [86], [87]), FLNA (neuronal migration [88]), and TUBB3 (neuronal migration [89] [90]). Glutamine synthetase (also known as glutamate-ammonia ligase), which protects the central nervous system by detoxifying glutamate [91], also appears in our list of salivary proteins that have been identified in exosomes. Of note, the beta and delta hemoglobin chains, commonly found in exosomes [73], show a significant positive correlation with cognitive scores. The hemoglobin beta and delta chains have been shown to give rise to peptides involved in enhancing learning and memory [92]. Thus, we suggest that a possible mechanism explaining our current findings may be the existence of a pathway connecting salivary proteins to cognitive function through the shuttle of salivary exosomes to the central nervous system.

Exosomes released by one cell have been shown to influence the response of another cell to an external stress by providing the recipient cell with resistance to the external stressor [93]. Eldh et al. have shown that exosomes released by cells exposed to a stressor, for example oxidative stress, can mediate a protective signal to another cell, making the recipient cell more tolerant to an oxidative process and subsequent cell death [93]. Accordingly, the salivary glands are known to be impacted by HIV disease [94], and therefore salivary gland cells are likely to be exposed to HIV-induced proteolytic stress. We therefore propose that salivary gland cells may be capable of releasing exosomes containing protein and RNA that can mediate a tolerizing effect to proteolytic stress in distant cells, including brain cells. Exosomes were recently identified in human CSF [95], and further research will be needed to explore the possibility that salivary exosomes contribute to the CSF exosome pool.

Our finding that salivary IL1RA has a significant negative correlation with cognitive scores in HIV^+^ subjects seems counterintuitive, as the deleterious role of IL-1 in the central nervous system has been firmly established in experimental models [63], [96]. However, Rimaniol et al. have demonstrated an imbalance between IL-1 and IL1RA in the CSF of HIV^+^ patients, noting high IL1RA concentrations, but undetectable levels of IL-1 [97]. Thus, our data showing a significant negative correlation of salivary IL1RA with cognition may be a reflection of elevated IL1RA levels in the CSF of HIV^+^ subjects. Since blood-borne IL1RA has been shown to cross the blood-brain barrier [98], [99], it is plausible that salivary IL1RA may translocate from the oral cavity to the brain during HIV infection to add to the imbalance between IL1RA and IL-1 and disrupt protein homeostasis. Transgenic mice over-expressing IL1RA in the brain have been shown to have impairments in the formation of long-term hippocampal-dependent memory [100], [101], and IL1RA protein aggregates have been identified in Alzheimer's disease plaques and tangles, and in the temporal cortex of patients with frontotemporal lobe dementia [102]. Wang et al. have suggested that the basal activity of the IL-1 receptor regulates the normal function of the ubiquitin-proteasome system and autophagy, and that elevated IL1RA activity could lead to the accumulation of misfolded proteins [103]. Thus, the proteins we identified that are involved in protein quality control, and that have positive correlations with cognitive scores, such as VCP, UBA1, and CANX, may represent a protective response to IL1RA-induced disruption of proteolysis. This hypothesis is consistent with the concept that defective protein quality control underlies HAND pathogenesis [72].

Limits of this study include the fact that all of the HIV^+^ subjects, except one, had a history of exposure to antiretroviral medications or were taking cART at the time of the study (Table 1). Therefore, we cannot say for certain that the observed proteomic and cognitive results are due solely to HIV-infection. Evaluation of the salivary proteome and cognitive scores in the HIV^+^ group does not distinguish patterns that may be due to cART exposure from those that are associated solely with chronic HIV infection. Future studies are required to decouple viral infection from cART in influencing proteomic and cognitive results. In addition, the HIV^−^ group was using significantly more methamphetamine/amphetamine than the HIV^+^ group (Table 1). Methamphetamine is known to cause proteasome inhibition and disruption of protein homeostasis [104] , so it is possible that any correlations that might exist between salivary proteins and cognitive scores in the HIV^−^ heroin users is disrupted by methamphetamine use. This could also possibly explain the more heterogenous nature of the HIV^−^ group in terms of methadone treatment response. We recognize a need to determine if HIV^+^, non-drug addicted subjects demonstrate significant correlations between salivary proteins and cognitive scores, however considering that HIV^−^, non-drug addicted subjects do not demonstrate saliva proteome DSST correlations, this leads us to believe that HIV is at least a major contributing factor to this result, and to what extent the additional stresses of drug abuse/treatment play a contributing role is yet to be determined, but presents avenues of further investigation. Lastly, our HIV^+^ group contained only males, which may have also contributed to the more homogeneous nature of the HIV^+^ group; however, limiting the HIV^−^, heroin-addicted group to only males (6) did not noticeably reduce the heterogeneity in methadone treatment responses (data not shown), so we doubt that gender was a major contributing factor. There is a need to determine if HIV^+^, heroin-addicted and non-drug addicted females demonstrate correlations between salivary proteins and cognition.

In conclusion, we have identified 58 salivary proteins that demonstrate significant correlations with cognitive scores in HIV^+^ heroin addicts, with only one protein, IL1RA, showing a negative correlation. These results were not found in HIV^−^ heroin addicts, or HIV^−^ non-addicts. A subset of these proteins appears sensitive to methadone treatment but the majority was independent of treatment. We propose a novel hypothesis that discrete packets of salivary proteins in the form of exosomes may be produced by the salivary glands and/or oral epithelium in response to HIV infection, and that these exosomes are capable of modulating brain function. Since exosomes were recently discovered in human cerebrospinal fluid (CSF) [95], it may be possible to design experiments to determine if salivary exosomes contribute to the CSF exosome pool. Furthermore, we propose that IL1RA, originating in the oral cavity, may translocate to the brain during HIV infection, disrupt protein homeostasis, and contribute to the pathogenesis of HAND. More research needs to be carried out to test these novel hypotheses.

## Supporting Information

Table S1 **Table of measured DSST scores for each participant and time point.**
(PDF)

Table S2 **List of peptide identifications and corresponding mass spectrometry metrics, probability values, and quantitative information for each analysis which support the reported protein identification and quantification.**
(XLSX)

Table S3 **List of protein identifications and quantitative information for each analysis.**
(XLSX)

Table S4 **List of specific salivary proteins from our current study that correlate with DSST scores that overlap with proteins that are previously shown to be modulated by morphine and other drugs of abuse.**
(PDF)

Table S5 **List of specific salivary proteins with correlations with DSST scores and proteins identified in prior proteomic studies related to HIV infection.**
(PDF)

Table S6 **List of 91 and 69 proteins from HIV+ and HIV- participants respectively with significant differential abundance at 35/36 days of methadone treatment.**
(XLSX)
