# Supplementary material for: Proteomic Analysis of Saliva in HIV-Positive Heroin Addicts Reveals Proteins Correlated with Cognition
Source: PLoS One. 2014 Apr 9;9(4):e89366. doi: 10.1371/journal.pone.0089366 (PMC3981673; doi:10.1371/journal.pone.0089366)
Supplement: Table S1 — Table of measured DSST scores for each participant and time point. (PDF) [file pone.0089366.s001.pdf]

Table S1. DSST* Scores Across Saliva Collection Time Points					
HIV$^-$ Heroin Addicts					
Subject #	1	2	3	4	5
1	46	49	55	62	61
2	31	12	32	29	30
3	61	62	63		
4	44	25	31		
5	28	35	27	27	28
6	65	61	59	67	73
7	51	38	48	56	
8	35	30	24	22	48
9	19	42	34	39	45
10	21	23	24	30	25
11	39	20	34		
HIV$^+$ Heroin Addicts					
1	38			42	37
2	21	32	19	20	31
3	47	43	55	58	47
4	35	33	43	45	43
5	21	21	26	16	30
6	26	29	31	23	33
7	35	40	25	35	38
8	38	53	46	33	
Non-Heroin, HIV$^-$					
1	46	49			
2	12	18			
3	44	34			
4	22	21			
5	49	54			
6	43	28			
7	21	21			
8	65	57			
9	45	48			
10	49	34			
11	18	43			

*DSST = Digit Symbol Substitution Test. The DSST was administered on the day of each saliva sample collection. There is not an agreed-upon threshold for normality for the DSST score, but lower DSST scores indicate worse performance (Rosano et al. J Am Geriatric Soc 2008; 56: 1618-1625).
