# Supplementary material for: Proteomic Analysis of Saliva in HIV-Positive Heroin Addicts Reveals Proteins Correlated with Cognition
Source: PLoS One. 2014 Apr 9;9(4):e89366. doi: 10.1371/journal.pone.0089366 (PMC3981673; doi:10.1371/journal.pone.0089366)
Supplement: Table S4 — List of specific salivary proteins from our current study that correlate with DSST scores that overlap with proteins that are previously shown to be modulated by morphine and other drugs of abuse. (PDF) [file pone.0089366.s004.pdf]

Table S4. Salivary proteins from Table 2 that overlap with proteins that are modulated in brain tissues by drugs of abuse (adapted from Wang et al. 2011)[1]				
UniProt	Protein name	Gene symbol	Drugs	Brain tissues (ref)
Q13509	Tubulin, beta 3 class III	TUBB3	Marijuana/ Morphine	Rat hippocampus[2]/rat primary striatal neuronal cell cultures[3]
O75083	WD repeat domain 1	WDR1	Morphine	Rat cerebral cortex, hippocampus, and striatum[4]
Q96ML2	Vimentin	VIM	Morphine	African Green Monkey CSF[5]
P15104	Glutamate-ammonia ligase	GLUL	MA/Cocaine/ Morphine	Rat amygdala[6]/rhesus monkey nucleus accumbens[7]/mouse hippocampus[8]
P26641	Eukaryotic translation elongation factor 1 gamma	EEF1G	MA/Cocaine	Rat cortex[9]/human astrocyte cultures[10]
P02042	Hemoglobin, delta	HBD	Morphine	African Green Monkey CSF[5]
P29401	Transketolase	TKT	Cocaine/Morphine/ Nicotine	Rat nucleus accumbens[11]/rat brains[12]/rat brains[13]
P08238	Heat shock protein 90kDa alpha (cytosolic), class B member 1	HSP90AB1	MA/Morphine	Rat cortex[9]/rat primary striatal neuronal cell cultures[3]
Q549N7	Hemoglobin subunit beta	HBB	Morphine	African Green Monkey CSF[5]
Abbreviations: MA = methamphetamine/amphetamine, CSF = cerebrospinal fluid				

1. Wang, J., Yuan, W. & Li, M.D. Genes and pathways co-associated with the exposure to multiple drugs of abuse, including alcohol, amphetamine/methamphetamine, cocaine, marijuana, morphine, and/or nicotine: a review of proteomics analyses. *Molecular neurobiology* **44**, 269-286 (2011).
2. Quinn, H.R., *et al.* Adolescent rats find repeated Delta(9)-THC less aversive than adult rats but display greater residual cognitive deficits and changes in hippocampal protein expression following exposure. *Neuropsychopharmacology : official publication of the American College of Neuropsychopharmacology* **33**, 1113-1126 (2008).
3. Bodzon-Kulakowska, A., *et al.* Proteomic analysis of striatal neuronal cell cultures after morphine administration. *Journal of separation science* **32**, 1200-1210 (2009).
4. Bierczynska-Krzysik, A., *et al.* Proteomic analysis of rat cerebral cortex, hippocampus and striatum after exposure to morphine. *International journal of molecular medicine* **18**, 775-784 (2006).
5. Brown, J.N., *et al.* Morphine produces immunosuppressive effects in nonhuman primates at the proteomic and cellular levels. *Molecular & cellular proteomics : MCP* **11**, 605-618 (2012).
6. Iwazaki, T., McGregor, I.S. & Matsumoto, I. Protein expression profile in the amygdala of rats with methamphetamine-induced behavioral sensitization. *Neuroscience letters* **435**, 113-119 (2008).

7. Tannu, N.S., Howell, L.L. & Hemby, S.E. Integrative proteomic analysis of the nucleus accumbens in rhesus monkeys following cocaine self-administration. *Molecular psychiatry* **15**, 185-203 (2010).
8. Moron, J.A.*, et al.* Morphine administration alters the profile of hippocampal postsynaptic density-associated proteins: a proteomics study focusing on endocytic proteins. *Molecular & cellular proteomics : MCP* **6**, 29-42 (2007).
9. Kobeissy, F.H.*, et al.* Psychoproteomic analysis of rat cortex following acute methamphetamine exposure. *Journal of proteome research* **7**, 1971-1983 (2008).
10. Reynolds, J.L.*, et al.* Proteomic analysis of the effects of cocaine on the enhancement of HIV-1 replication in normal human astrocytes (NHA). *Brain research* **1123**, 226-236 (2006).
11. del Castillo, C.*, et al.* Proteomic analysis of the nucleus accumbens of rats with different vulnerability to cocaine addiction. *Neuropharmacology* **57**, 41-48 (2009).
12. Kim, S.Y.*, et al.* Proteomic analysis of phosphotyrosyl proteins in morphine-dependent rat brains. *Brain research. Molecular brain research* **133**, 58-70 (2005).
13. Hwang, Y.Y. & Li, M.D. Proteins differentially expressed in response to nicotine in five rat brain regions: identification using a 2-DE/MS-based proteomics approach. *Proteomics* **6**, 3138-3153 (2006).
