# Supplementary material for: Proteomic Analysis of Saliva in HIV-Positive Heroin Addicts Reveals Proteins Correlated with Cognition
Source: PLoS One. 2014 Apr 9;9(4):e89366. doi: 10.1371/journal.pone.0089366 (PMC3981673; doi:10.1371/journal.pone.0089366)
Supplement: Table S5 — List of specific salivary proteins with correlations with DSST scores and proteins identified in prior proteomic studies related to HIV infection. (PDF) [file pone.0089366.s005.pdf]

Table S5. Overlap of salivary proteins from Table 2 and brain proteins identified in HIV-1 infected subjects by proteomic analysis.

UniProt	Protein name	Gene symbol	Brain tissue	Study description (ref)
P16152	Carbonyl reductase 1	CBR1	Frontal cortex (Human)	Increased 2 fold in HAD vs. HIV+ without dementia (Zhou 2010)
P08107	Heat shock 70kDa protein 1A	HSPA1A	Frontal neocortex synaptosomes (Human)	Increased 2.00 fold in HIV+ synaptosomes with high immunoproteosomes (Gelman 2010)
P21333	Filamin A, alpha	FLNA	CSF (Human)	Identified in CSF of HIV-infected subjects (Angel 2012)
Q96ML2	Vimentin	VIM	CSF (Human)	High confidence identification in CSF of HIV-infected subjects (Rozek 2007)
P15104	Glutamate-ammonia ligase	GLUL	Frontal cortex (Human)	Increased 1.85 fold in HAD vs. HIV+ without dementia (Zhou 2010)
P02511	Crystallin, alpha B	CRYAB	Frontal neocortex synaptosomes (Human)	Decreased - 2.11 fold in HIV+ synaptosomes with high immunoproteosomes (Gelman 2010)
P22314	Ubiquitin-like modifier activating enzyme 1	UBA1	Frontal neocortex synaptosomes (Human)	Increased 2.02 fold in HIV+ synaptosomes with high immunoproteosomes (Gelman 2010)
Q549N7	Hemoglobin subunit beta	HBB	CSF (Human)	Identified in CSF of HIV-infected subjects (Angel 2012)
	? beta tubulin			(Gelman 2010)
	? alpha tubulin			(Gelman 2010)
